# Supplementary material for: Prime editing-mediated correction of the CFTR W1282X mutation in iPSCs and derived airway epithelial cells
Source: PLoS One. 2023 Nov 29;18(11):e0295009. doi: 10.1371/journal.pone.0295009 (PMC10686454; doi:10.1371/journal.pone.0295009)
Supplement: S1 Appendix — (DOCX) [file pone.0295009.s001.docx]

**S1 Fig.** **Optimization of prime editing strategy in HEK 293T cells.**


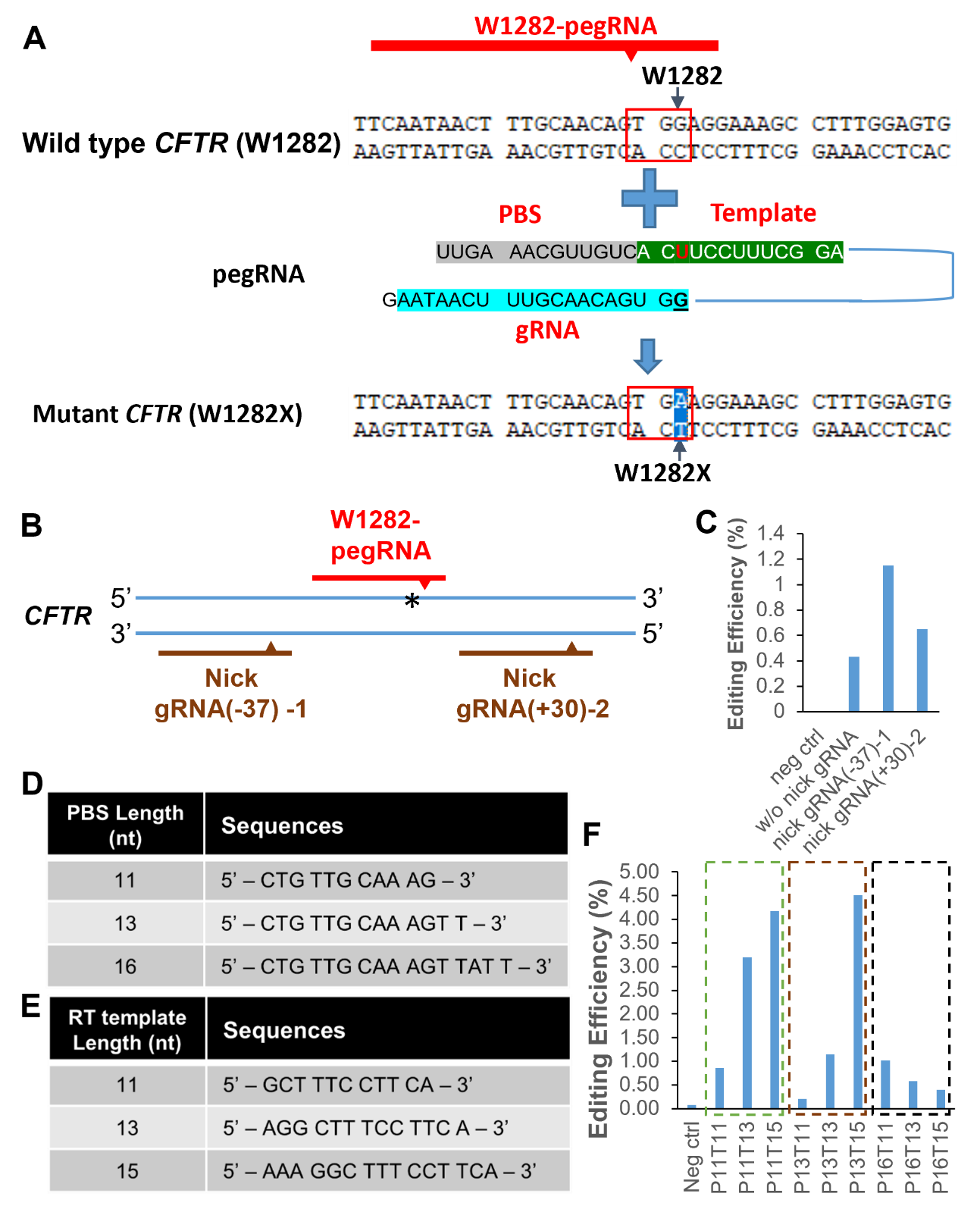


**S2 Fig. Sib selection and Sanger sequencing analyses of PE-iPSC clones.**

**
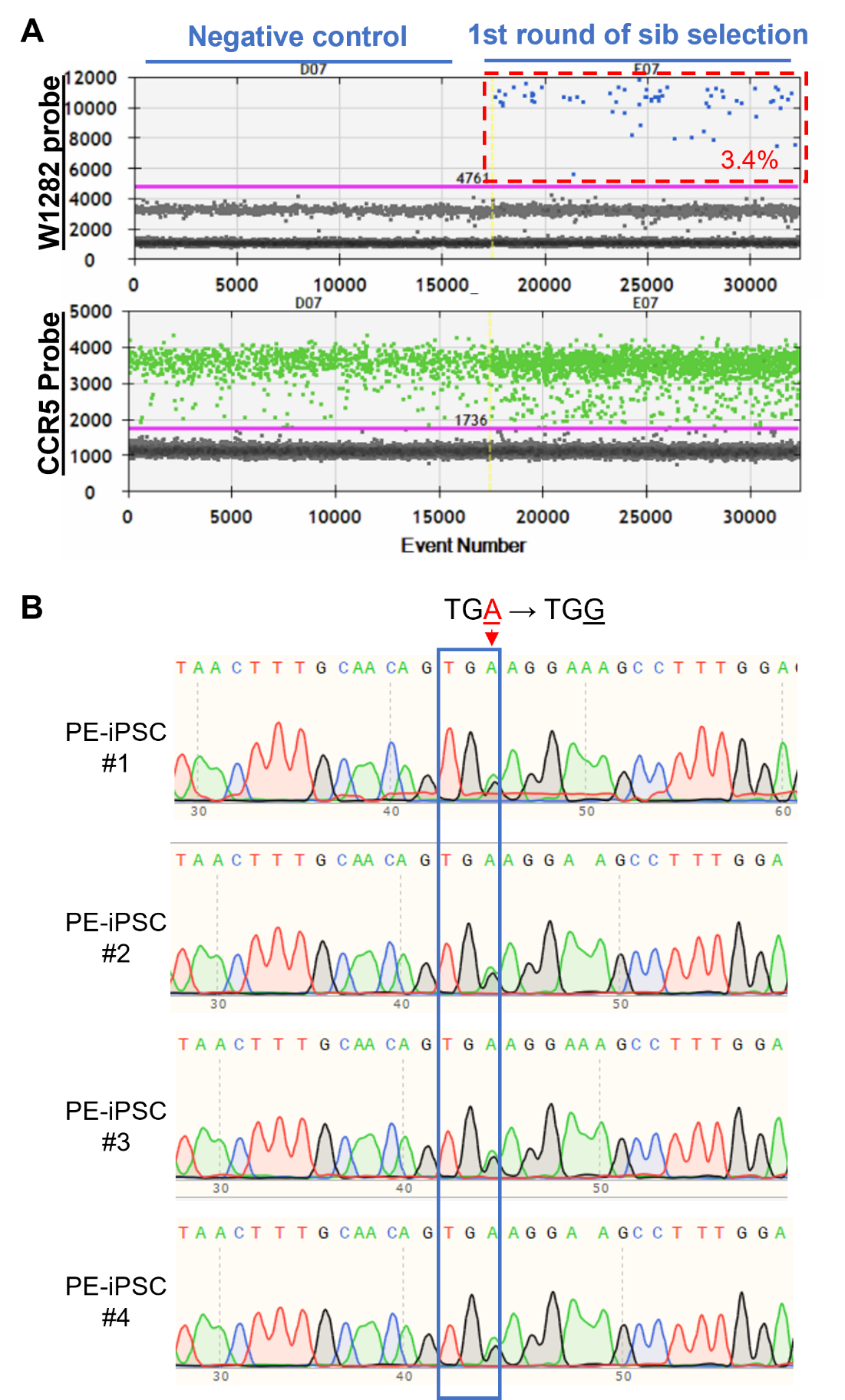
**

**S3 Fig. HDAd can effectively transduce iPSC-derived airway epithelial cells.**

**
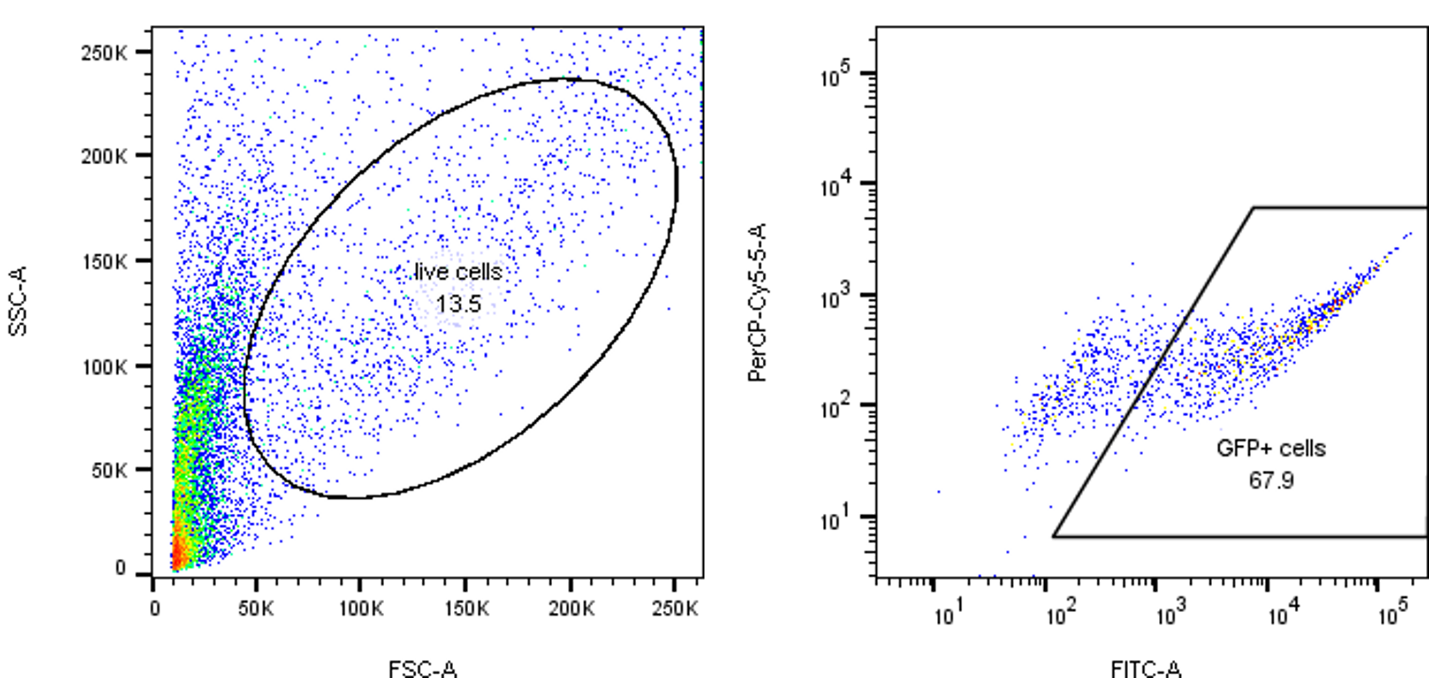
**

**S4 Fig. Ussing chamber assays of differentiated CFTR W1282X airway epithelium corrected by HDAd-delivered prime editing.**

**
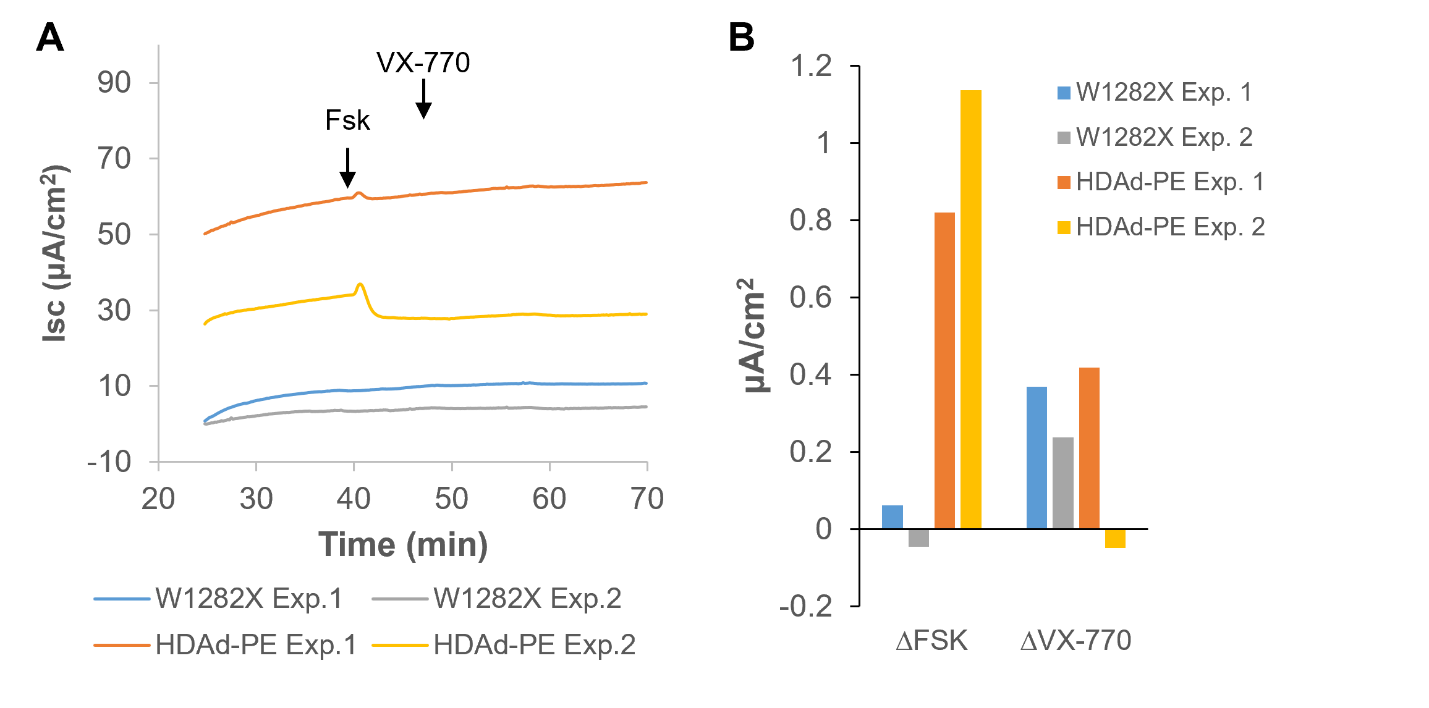
**

# Supporting Figure legends

**S1 Fig. Optimization of prime editing strategy in HEK 293T cells.**

1. The W1282-pegRNA and the DNA target. The W1282-pegRNA is designed to convert the wildtype W1282 codon to X1282. PBS, primer binding site. In addition to the tracrRNA (solid blue line) that binds to the nCas9, each pegRNA contains three key functional components – the crRNA, PBS, and template. crRNA brings the prime editor to the target site. PBS, primer binding site.
2. Schematic of the two nick gRNAs adjacent to the W1282 codon.
3. Quantification of ddPCR analyses on the efficiencies of nick gRNAs in helping edit the W1282 codon.
4. PBS sequences that had been tested.
5. RT template sequences that had been tested.
6. Quantification of ddPCR analysis on W1282 editing efficiencies of pegRNAs with different PBS and RT lengths. Note that on average pegRNAs with an 11-nt PBS have the highest editing efficiencies and pegRNAs with a 15-nt RT template have the highest efficiencies. Based on these observations, the P11T15 pegRNA had been selected for further use.

**S2 Fig. Sib selection and Sanger sequencing analyses of PE-iPSC clones.**

1. ddPCR analysis indicates a 10-fold enrichment of the corrected allele (from 0.36% to 3.4%) after one round of sib selection of the iPSCs analyzed in Figure 1D. Negative control, W1282X-iPSCs not transfected with prime editing machinery. The W1282 probe only recognizes corrected *CFTR* alleles. The CCR5 probe is used as a copy number control for the input genomic DNA.
2. Sequencing analyses of PCR fragments containing the W1282 codon amplified from genomic DNA of four independent PE-iPSC clones. Blue box, the W1282X codon. Note that INDELs were observed in neither the edited allele nor the non-edited allele.

**S3 Fig. HDAd can effectively transduce iPSC-derived airway epithelial cells.** Flow cytometry analysis of a representative sample of iPSC-derived airway epithelial cells 72 hours after HDAd transduction.

**S4 Fig. Ussing chamber assays of differentiated CFTR W1282X airway epithelium corrected by HDAd-delivered prime editing.**

1. Ussing chamber assays that measure short circuit current (Isc) traces of W1282X-iPSC-derived airway epithelia that were corrected by HDAd-delivered prime editing (HDAd-PE). Unedited airway epithelia (W1282X) were used as control. FSK, forskolin.
2. Quantification of the Isc traces in **(A)**.

# Supporting movie legends

**PE organoid FIS assay.** Swelling of the lung organoids differentiated from PE-iPSCs over 8 hours after stimulation by 10 μM forskolin.

**W1282X organoid FIS assay.** Swelling of the lung organoids differentiated from W1282X-iPSCs over 8 hours after stimulation by 10 μM forskolin.

**S1 Table. Sequences of pegRNAs, nick gRNAs, ddPCR primers, and probes.**

| **pegRNA** | **Spacer Sequences** | **3' Extention Sequences** | **Edits** |
| --- | --- | --- | --- |
| W1282X-P11T11 | 5' – GCA ATA ACT TTG CAA CAG TGG - 3' | 5' – GCT TTC CT**t** CAC TGT TGC AAA G - 3' | G to A |
| W1282X-P11T13 | 5' – GCA ATA ACT TTG CAA CAG TGG - 3' | 5' – AGG CTT TCC T**t**C ACT GTT GCA AAG - 3' | G to A |
| W1282X-P11T15 | 5' – GCA ATA ACT TTG CAA CAG TGG - 3' | 5' – AAA GGC TTT CCT **t**CA CTG TTG CAA AG - 3' | G to A |
| W1282X-P13T11 | 5' – GCA ATA ACT TTG CAA CAG TGG - 3' | 5' – GCT TTC CT**t** CAC TGT TGC AAA GTT - 3' | G to A |
| W1282X-P13T13 | 5' – GCA ATA ACT TTG CAA CAG TGG - 3' | 5' – AGG CTT TCC T**t**C ACT GTT GCA AAG TT - 3' | G to A |
| W1282X-P13T15 | 5' – GCA ATA ACT TTG CAA CAG TGG - 3' | 5' – AAA GGC TTT CCT **t**CA CTG TTG CAA AGT T - 3' | G to A |
| W1282X-P16T11 | 5' – GCA ATA ACT TTG CAA CAG TGG - 3' | 5' – GCT TTC CT**t** CAC TGT TGC AAA GTT ATT - 3' | G to A |
| W1282X-P16T13 | 5' – GCA ATA ACT TTG CAA CAG TGG - 3' | 5' – AGG CTT TCC T**t**C ACT GTT GCA AAG TTA TT - 3' | G to A |
| W1282X-P16T15 | 5' – GCA ATA ACT TTG CAA CAG TGG - 3' | 5' – AAA GGC TTT CCT **t**CA CTG TTG CAA AGT TAT T - 3' | G to A |
| X1282W-P11T15 | 5' – GCA ATA ACT TTG CAA CAG TGA - 3' | 5' – AAA GGC TTT CCT **c**CA CTG TTG CAA AG - 3' | A to G |
|  |  |  |  |

| **Nick sgRNA** | **Spacer Sequences** |
| --- | --- |
| Nick gRNA(-37)-1 | 5' – GCC CAA GAC ACA CCA TCG ATC - 3' |
| Nick gRNA(+30)-2 | 5' – GTA AGT CCT TTT GCT CAC CTG - 3' |
|  |  |
| **ddPCR Primers** | **Sequences** |
| W1282X-f1 | 5' – GAG ACT ACT GAA CAC TGA AGG AGA AA - 3' |
| W1282X-r2 | 5' – CTG TGG TAT CAC TCC AAA GGC TT - 3' |
| CCR5-f | 5' – TAC ATC GGA GCC CTG CCA - 3' |
| CCR5-r | 5' – ATG TCA GTC ATG CTC TTC AGC CT - 3' |
|  |  |
| **ddPCR probes** | **Sequences** |
| W1282-FAM-TM | 5' – caa cag tgG agg aaa - 3' |
| X1282-FAM-TM | 5' – caa cag tgA agg aaa - 3' |
| CCR5-probe-HEX | 5' – CTC CGC TCT ACT CAC TGG TGT TCA TC - 3' |
|  |  |
| **PCR Primers** | **Sequences** |
| CFTR W1282-F | 5' – TTG GGA AGA ACT GGA TCA GG - 3' |
| CFTR W1282-r | 5' – CCA AGG CTC CCA CTG TAA AT - 3' |
